# Flexible and Physically Unclonable Function Anti-Counterfeiting Labels via Multi-Level Dynamic Structural Color Encryption

**DOI:** 10.3390/ma19071428

**Published:** 2026-04-02

**Authors:** Junzhe Lin, Min Zhao, Xueqing Zhu, Ruohan Guo, Dan Guo, Tianrui Zhai

**Affiliations:** Department of Physics and Optoelectronic Engineering, Beijing University of Technology, Beijing 100124, China; opticsljz@163.com (J.L.); zhaomin2023@emails.bjut.edu.cn (M.Z.); zxqzxqdd92@163.com (X.Z.); gruohan@emails.bjut.edu.cn (R.G.); dguo@bjut.edu.cn (D.G.)

**Keywords:** physically unclonable functions, structural color, flexible, photonic crystals, dynamic encryption

## Abstract

Physically unclonable functions (PUFs) are critical security primitives used in authentication and cryptographic key generation. Among these, structural color-based PUFs offer distinct advantages, including fade resistance and the ability to conceal multi-dimensional information. However, current fabrication methods rely heavily on wet processes and laser ablation. Consequently, there is a significant need for flexible PUF labels capable of being produced through a facile and dry process. Here, we present stress-relief modulated photonic crystal PUF labels designed for multi-level dynamic encryption. We achieve random patterning of nanograting-based photonic crystals by leveraging curved pinning edge-induced interruptions and the uncontrolled bulking of the polymeric elastomer due to the uneven adhesion force from the tape. Using artificial intelligence-based deep learning algorithms, we authenticate the labels by extracting structural color, brightness, and saturation, which are determined by the grating periodicity, depth, and orderliness of each pixel. Furthermore, we integrated these photonic crystal patterns with dynamically modulated optical erasure to extend encryption capacity from the spatial to the temporal dimension. We anticipate this approach will enable advanced wearable anti-counterfeiting labels and multi-level digital encryption systems.

## 1. Introduction

Counterfeit and substandard products have become a pressing global issue, impacting sectors ranging from consumer goods to pharmaceuticals and high-tech industries. These products threaten public health, undermine economic stability, and pose risks to national security [1,2,3,4,5]. Over the past few decades, significant research has focused on anti-counterfeiting strategies, driving the rapid advancement of encryption technologies such as watermarking, holography, and QR/barcodes. While these methods offer improved concealment and multi-channel security, they remain hindered by limitations like the degradation of fluorescent dyes, susceptibility to replication, and complex decryption processes [6,7,8]. Consequently, ideal security labels meet the four essential requirements: (1) high environmental durability, (2) low-cost manufacturing, (3) dynamic, unclonable encryption, and (4) accessible readout mechanisms. The encryption strategy based on structural color provides an effective and facile approach for optical security. Recent advances have enabled high-security and easily readable encryption by incorporating angle-dependent responses, fluorescence emission, circularly polarized luminescence, and other multi-dimensional optical channels [9,10,11]. Nevertheless, it remains a critical challenge to simultaneously achieve ultra-high security, reliable readability, and dynamic encryption via a simple fabrication process.

In recent years, Physical Unclonable Functions (PUFs) have garnered considerable interest as a robust solution. PUFs exploit intrinsic, stochastic variations in the manufacturing process to generate unique keys derived solely from physical structure rather than external inputs [12,13,14]. Optical PUF systems, in particular, offer high-dimensional encryption, as their responses are highly sensitive to minute structural changes—making them virtually impossible to clone. Concurrently, advances in deep learning have enabled Artificial Intelligence (AI)-powered authentication, offering a practical solution for verifying these complex signatures [15,16,17,18]. Although numerous PUF labels have been fabricated using metals [19,20,21,22], semiconductors [23,24,25,26], photonic structures [27,28], DNA [29,30,31], and hydrogels [32,33]—often via dip-coating [34], inkjet printing [35], or self-assembly [36,37]—barriers to widespread adoption remain. Integrating PUF technology with deep learning presents a clear opportunity for stable, dynamic optical security labels. For instance, there have been numerous reports on nanofabrication technologies, such as nano-lithography, nano-imprinting, colloidal self-assembly, and inkjet printing, which are employed to design optical structures for implementing anti-counterfeiting encryption functions based on physical unclonable functions (PUF) [38,39,40]. However, these preparation procedures generally rely on complex wet processing or laser ablation assistance. Hence, the development of PUF labels with dynamic properties and anti-counterfeiting characteristics through simple, low-cost strategies remains highly desirable.

In this study, we present a novel strategy for PUF labels featuring multi-level encryption capabilities. By employing the stochastic formation of grating arrays—caused by the uncontrolled bulking of the polymeric elastomer due to the uneven adhesion force from the tape—as the encoding medium, we fabricated Photonic Crystal Physical Unclonable Labels (PCPUL) on flexible polymer substrates. We authenticated these labels using artificial intelligence-based deep learning algorithms to extract structural color, brightness, and saturation—properties governed by the local grating periodicity, depth, and orderliness. Furthermore, the orthogonal integration of distinct photonic crystal patterns, combined with polymer swelling-induced optical erasure, extended the encryption capacity from the spatial to the temporal dimension.

## 2. Materials and Methods

### 2.1. Preparation of PDMS Flexible Film

Polydimethylsiloxane (PDMS) soft substrates are prepared by mixing the PDMS oligomer and curing agent in different mass ratios (Dow Corning, Sylgard 184). The softness of the formed PDMS elastomer increases with the PDMS oligomer ratio. The mixture is thoroughly stirred and centrifuged at 3000 rpm for 3 min to remove bubbles. The solution was spin coated (200 rpm, 5 min) on a glass slide, and was cured in an oven at 80 °C for 2 h.

### 2.2. Preparation of PCPUL

The PDMS flexible film was quantitatively stretched on a glass substrate and fixed at both edges with adhesive tape. The pre-patterned mask was then attached to the stretched film. The assembly was treated in a PLUTO-T plasma system for 120 s at 150 W. The structural color of the directionally stretched, plasma-treated PDMS is attributed to the formation of a surface diffraction grating. Plasma treatment oxidizes the PDMS surface to form a rigid silicate layer; the mechanical mismatch between this rigid layer and the compliant bulk induces stress relaxation, leading to the generation of periodic wrinkle patterns.

### 2.3. PUF Randomness Assessment

The μ parameter is the average value of each statistical distribution (Hamming distance, correlation coefficient, entropy varying with the sample). It is used to quantify the overall characteristics of the data. In the randomness assessment, the μ value of ideal random data has specific expectations. The Hamming distance μ = 0.5, the correlation coefficient μ = 0, and the entropy value μ = 8.

$$\mu =\left(1∕n\right)*\Sigma_{i}x_{i}\;(i=1,2,3\ldots n)$$

The correlation coefficient (Correlation Coefficient) is used to measure the degree of linear correlation between two variables, and it can detect periodic data or patterns in the sequence. To calculate the correlation coefficient, parameters such as the expected value, variance, and covariance of the sample are required. Based on the calculation results, the correlation coefficients of the three-channel information all fall within the range of extremely weak correlation or no correlation.

$$\rho \left(X,Y\right)=\mathrm{cov}\left(X,Y\right)/\left(\sigma_{X}*\sigma_{Y}\right)=E\left[\left(X-\mu_{X}\right)*\left(Y-\mu_{Y}\right)\right]/\left(\sigma_{X}*\sigma_{Y}\right)$$

$$E\left(X\right)=\mu_{X}=\frac{1}{n}*\sum_{i=1}^{n}X_{i}$$

$$\sigma_{X}^{2}=\frac{1}{n}\sum_{i=1}^{n}{\left(X_{i}-\mu_{X}\right)}^{2}$$

$$\mathrm{cov}\left(X,Y\right)=\frac{1}{n}\sum_{i=1}^{n}\left(X_{i}-\mu_{X}\right)*\left(Y_{i}-\mu_{Y}\right)$$

The Hamming Distance is defined as the number of differing characters at corresponding positions between two equal-length strings. Here, we normalize the Hamming Distance: the original Hamming Distance is divided by the length of the string. Therefore, for two completely random binary strings: the ideal Hamming Distance value is 0.5. Based on the calculation results, the normalized Hamming Distance of the three-channel information is close to 0.5, which proves the existence of good randomness.

$$H\left(X,Y\right)=\Sigma_{i}\left(X_{i}\neq Y_{i}\right)$$

Entropy is used to measure the uncertainty or randomness of information. Here are the steps for calculating entropy:

(1) Data preprocessing: Convert the original data into numerical form and normalize it to the range of 0–255. (2) Calculate probability distribution: Calculate the probability of each numerical value (normalized probability $P\left(X_{i}\right)$). (3) Calculate entropy: For each non-zero probability $P\left(X_{i}\right)$, calculate $P\left(X_{i}\right)*\log\left(P\left(X_{i}\right)\right)$, then sum them up and take the negative.

$$Entropy(X)=-\sum_{i=1}^{n}P\left(X_{i}\right)*\log\left(P\left(X_{i}\right)\right)$$

The higher the entropy value, the greater the uncertainty of the data, that is, the better the randomness. For 8-bit data (0–255), the maximum entropy is 8. Based on the calculation results, the entropy values of the three channels are concentrated between 2 and 3, which proves that the data has good uncertainty.

### 2.4. PCPUL Dynamic Encryption Experiment

We put 10 mL of a toluene/xylene solution in a 20 mL spray dispensing bottle, and applied approximately 200 μL via spraying on the surface of the sample within the optical path system. After approximately 100 s, the structural color information gradually recovered. Heating can accelerate the recovery process.

### 2.5. Details of Encoding

First, we interactively read and intelligently processed the multi-format data; then calculated the three core indicators (Entropy, Hamming Distance, and Correlation Coefficient) and their μ parameters; next, we integrated the three dimensions for randomness assessment; finally, through standardized visualization and multi-format output, we presented the results to achieve a fully automated analysis process from data loading to the generation of the assessment report. At the end of the Supporting Information (Figure S13), we used blank group images to verify the accuracy and applicability of the code.

### 2.6. Deep Learning

Deep learning modeling was performed on the MATLAB (R2023a) platform based on the AlexNet convolutional neural network, and training samples were augmented via data augmentation. Firstly, representative structural color patterns were collected from specific angles to construct a PUF security label database, which were first resized to 512 × 512 pixels according to the pixel-region relationship, and the pattern filling density was calibrated to 0.8. Subsequently, image enhancement operations including rotation, scaling and random positioning were conducted, with 360° rotation at a step of 1° combined with random positioning, generating 360 images for each angle. In total, a deep learning dataset containing 360 × N_β_ images was constructed and divided into model training and identity authentication sets at a ratio of 5:1. Validation tests were carried out after each round of model training, and the recognition accuracy stabilized and converged to 1 after approximately 10 learning epochs.

## 3. Results and Discussion

### 3.1. Preparation and Encryption Strategy

**Figure 1:**
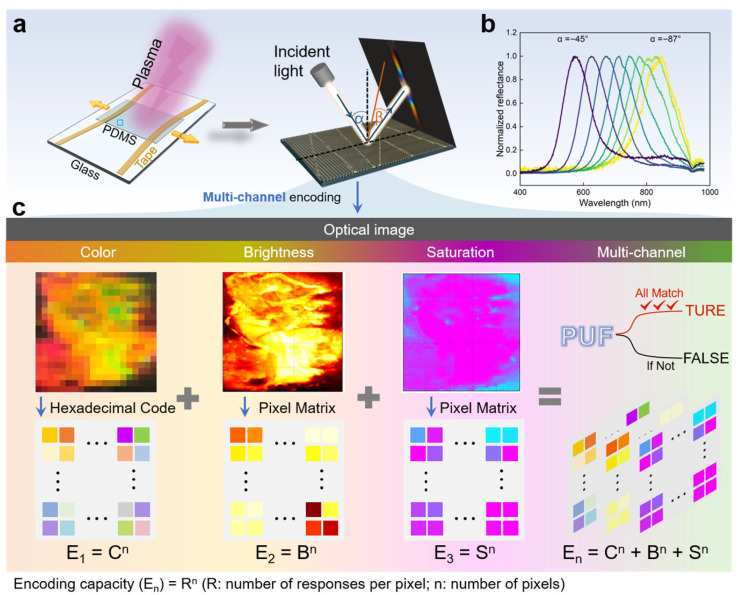
Design strategy of the multi-channel information-encrypted PCPUL. (**a**) Schematic illustration of the fabrication process. The left panel depicts grating arrays formation driven by directional stress relief; the curved tape edge interrupts the orientational formation of the nanograting arrays. The right panel displays the iridescence generated by the regionalized photonic crystal structure. (**b**) Angle-resolved reflection spectroscopy. (**c**) Multi-channel information encoding process of the PCPUL by extracting hexadecimal color codes, brightness pixel matrices, and saturation pixel matrices.

As illustrated in Figure 1a, the fabrication process began with a stretched PDMS film (see Section 2). The film was pinned by a non-uniformly adhesive and curved tape edge under the directional stretching. In the subsequent plasma treating progress, nano-gratings were generated stemming from the pinned edge, which serve as the prior stress relief sites. The microscopic ordering originated from the formation of grating arrays driven by directional stress relaxation [41]. Strain heterogeneity in stretched PDMS is caused by boundary constraints, Poisson’s effect, and inhomogeneous polymer cross-linking. During plasma treatment, such strain inhomogeneity together with non-uniform plasma energy density leads to spatial variations in the period and amplitude of surface micro-wrinkles, thus generating multicolor structural colors on PDMS [42]. Building on this, we introduced stochasticity through two pathways: (1) the multidirectional grating formation caused by the curved tape edge and (2) the stochastic buckling due to the uneven adhesion force between the pinned tape and the PDMS. These factors effectively integrated stochastic characteristics into the photonic crystal structure. As comparison, predictable structural color patterns are generated under uniform mechanical stretching (Figure S1). The presence of an ordered photonic crystal structure was confirmed via optical microscopy, visible iridescence under white light, and angle-resolved spectroscopy. The SEM images and angle-resolved reflection spectroscopy from the randomly selected region (approximately 0.5 cm × 0.5 cm) confirmed the successful formation of ordered photonic crystal structures (Figure 1b and Figure S2). Furthermore, random cracks tend to form during this process (Figure S3), affected by both the curing agent ratio, the stretching ratio, curved edges and the buckling instability. These randomly distributed cracks act as “scatters”, leading to the stochastic distribution of the saturation. These structural variations directly result in a random distribution of color, brightness, and saturation (Figure 1c). The inherent randomness stems from uncontrollable manufacturing variables, such as inhomogeneous fluctuations during stress release. Variations in experimental conditions—including stretching force and plasma parameters—ensured the PUF’s unclonability. Finally, we pixelated the structural color image to extract three-channel information: hexadecimal color codes, a brightness pixel matrix, and a saturation pixel matrix. These matrices formed the foundational encryption dataset E_n_ = C^n^ + B^n^ + S^n^. The security label thus produced a unique, unpredictable output, with verification returning “TRUE” only when pixel information matched across all three channels.

### 3.2. Information Patternization and Anti-Counterfeiting Database Construction

**Figure 2:**
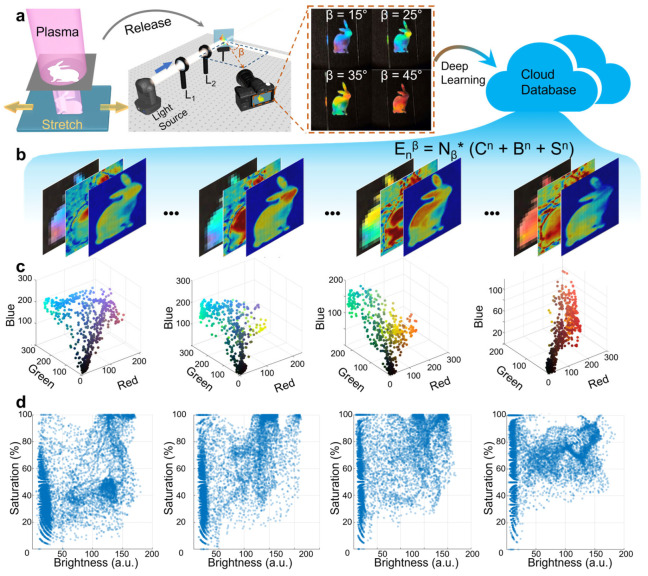
Structural color patterning, optical characterization, and encoding capacity. (**a**) Schematic of the patterning process and optical testing workflow. Images captured at various angles are processed using deep learning to construct a database for anti-counterfeiting applications. (**b**) The database is constructed by integrating information from three channels (color, brightness, and saturation). (**c**,**d**) Angle-dependent encoding capacity, illustrated by varying three-dimensional color space distribution (**c**), as well as the corresponding distributions of brightness and saturation (**d**).

Based on the three-channel encoding of the PCPUL, we further enhanced the encryption level by enriching the pattern’s boundaries and incorporating angle-dependent optical properties. Figure 2a showed the variability of the structural colors using a rabbit pattern, intensifying the stochasticity of the encrypted information, as edge effects significantly amplified structural randomness. To examine how boundary effects influence the randomness of ordered structures, we fabricated patterned samples with distinct geometric elements—including triangles, circles, squares, and rectangles of varying aspect ratios—using the tape-assisted random stress-release system (Figure S4). Under identical experimental conditions, clear differences in structural color are observed across the different patterns, confirming the significance of the edges. Consequently, the system gained two layers of security: angle dependence introduced an additional encryption dimension, and patterning reinforced the physical unclonability of the structural color. As shown in Figure 2b, extracting three-channel data from multiple angles effectively multiplied the encoding capacity of the database by a factor of N_β_, E_n_^β^ = N_β_ × E_n_, where N_β_ is constrained only by the resolution of the imaging system. Theoretically, this enabled enhancement in security compared to conventional labels. Figure 2c,d demonstrated this distinctiveness: data extracted at different angles revealed pronounced variations in color, brightness, and saturation. Based on this, we further calculated the statistical parameters of the data and analyzed their randomness (Figure S5), verifying the PUF characteristics.

### 3.3. Multidimensional Information Encryption and Statistical Characteristic Analysis

**Figure 3:**
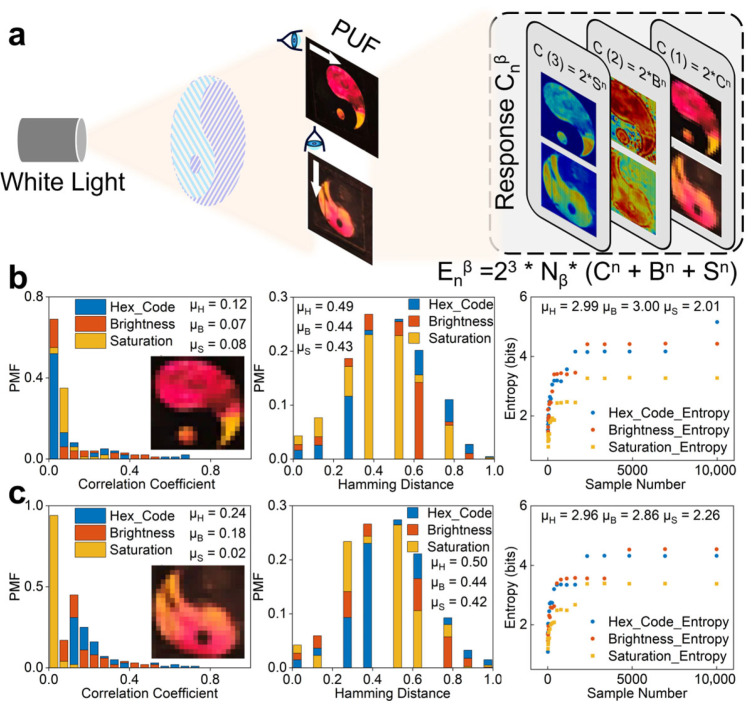
Multi-dimensional encryption and statistical characterization. (**a**) Schematic illustration of encoding capacity expansion by structural orientation grouping in a Tai Chi pattern. The white arrow indicates the view direction. (**b**,**c**) Quantitative randomness evaluation of PUF information across different structural orientations using statistical parameters, including information entropy, Hamming distance, and correlation coefficient.

Employing this facile and dry fabrication approach, we also demonstrate the view-angle dependent variation in the pattern via programmable treatments. Using two complementary patterns of the Tai Chi as masks, we successively stretched the PDMS film twice along two orthogonal directions, and obtained two sets of grating patterns with perfectly orthogonal orientations. The orientational dependence multiplied the cryptographic key complexity, providing an additional dimension for exponential growth in encoding capacity. As shown in Figure 3a, we integrated photonic crystal structures with varying orientations into the single label, allowing distinct structural color information to be extracted along two perpendicular directions (Figures S6 and S7). This effectively increased the encoding capacity by a factor of 2^3^, E_n_^β^ =2^3^ × N_β_ × E_n_. Alternatively, the encrypted information from one specific orientation group functioned as a preliminary security protocol. This layer served as a convenient and effective ‘handshake’ before the system engaged in the standard PUF-based interaction. Consequently, the verification mode could be flexibly switched based on the required security level. Moreover, the enhanced irregular curvature of the pattern’s edge enabled the calculated arithmetic average values of the correlation coefficient (0.02) and Hamming distance (0.50) to be both close to the ideal values (i.e., 0, 0.5) (Figure 3a,b), indicating the excellent uniformity, uniqueness, and randomness exhibited by the photonic crystal PUF labels (Figure S8).

### 3.4. Dynamic Encryption Strategy

**Figure 4:**
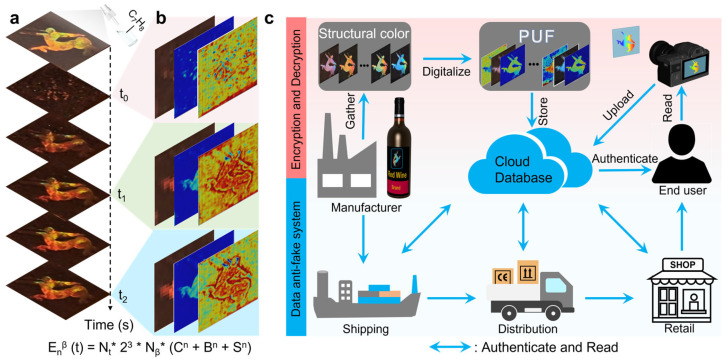
Time-dependent structural color distribution for expanded PUF encryption capacity. (**a**) Time-resolved photographs demonstrate the dynamical variation in the label information. (**b**) Extraction of multi-channel information—color, brightness, and saturation—at different time points to achieve dynamic encoding. (**c**) Demonstration of the PCPUL’s application in both public and personal anti-counterfeiting systems.

Furthermore, we introduced a temporal-dimension strategy alongside our spatial approach, leveraging the swelling behavior of flexible polymers to provide dynamic encryption capabilities. As shown in Figure 4a,b, exposure to toluene or xylene induced temporary swelling in the patterned film, causing the anti-counterfeiting information to shift or temporarily vanish (Figure S9). Upon solvent evaporation, the encrypted information gradually recovered to its original state within two minutes, which can be accelerated through heating treatment. Crucially, each frame captured during this reversible process contained a unique PUF encryption matrix, effectively enhancing the encoding capacity by a factor of N_t_, E_n_^β^(t) = N_t_ × 2^3^ × N_β_ × E_n_. To gain higher randomness, we made a wine product’s trademark pattern with complex contours to demonstrate security labels (Figures S10–S12). Statistical data demonstrated that each image frame can provide a unique PUF encryption matrix. Furthermore, due to the stochastic nature of the physical alterations during swelling, each solvent application generated a fresh key sequence, which can be further modulated by ambient temperature. This approach introduced non-deterministic, time-based encoding into the multi-dimensional encryption system, significantly increasing its resistance to decryption. Notably, after 50 repeated expansion cycles triggered by a small amount of atomized solvent (Supplementary Video S1), the samples showed no obvious structural damage or degradation of structural color information. Owing to the superior hydrophobicity of PDMS films, the samples exhibit excellent humidity stability under ambient conditions. Time-dependent dynamic encryption thus represented a powerful advancement toward the highest level of security in anti-counterfeiting materials.

Figure 4c outlined the practical implementation of the PCPUL for anti-counterfeiting and authentication. Using premium wine products as a case study, we demonstrated how the label’s patterning capability allowed for the seamless integration of encrypted information with the product logo. This approach preserved the esthetic integrity of the packaging while ensuring high-level security. The process began after manufacturing, where the structural color information of the security label was acquired. Artificial intelligence then processed the color, brightness, and saturation matrices to construct a deep-learning database. To ensure robust performance, the AI model iteratively refined its learning using images captured under various conditions, accounting for variables such as lighting and imaging angles. Finally, consumers can verify the product’s authenticity simply by capturing and uploading a photo of the label.

## 4. Conclusions

In conclusion, we have developed a flexible, structural color-based PUF label via a facile and dry fabrication process. By exploiting the stochastic nature of stress relief in plasma-treated polymers, we achieved high-entropy random patterning that serves as a robust cryptographic primitive. The resulting platform enables multi-dimensional encryption by extracting three-channel optical information (color, brightness, and saturation) and leveraging angle-dependent structural variations, thereby exponentially enhancing encoding capacity. In addition to static spatial encryption, a unique temporal dimension is incorporated through solvent-induced polymer swelling, which allows for dynamic key generation and enhances resistance to replay attacks. When integrated with deep learning-based authentication algorithms, the structurally colored PUF label demonstrates high accuracy and resilience against environmental interference. Unlike conventional structural color anti-counterfeiting approaches, this work successfully imparts a physically unclonable characteristic to structural color patterns through a simple fabrication process and encryption scheme. This work is anticipated to provide a low-cost and virtually unforgeable solution, which is highly promising for advanced wearable digital security applications.

## Data Availability

The original contributions presented in this study are included in the article/Supplementary Material. Further inquiries can be directed to the corresponding author.

## Supplementary Materials

The following supporting information can be downloaded at: https://www.mdpi.com/article/10.3390/ma19071428/s1, Figure S1: Schematic diagrams and structural color images of the two fabrication processes. Figure S2: Angle-resolved spectroscopy. Figure S3: Microscope images of samples prepared with different stretching ratios and polymer compositions. Scale bar is 10 μm. Figure S4: Structural color images of different graphic elements. Figure S5: Randomness assessment of color information in the rabbit image for β = 15°(a), β = 25°(b), β = 35°(c) and β = 45°(d). Figure S6: Structural color images from different observation directions. Figure S7: Three-dimensional color space distribution (a) and the corresponding distributions of brightness and saturation (b) of Tai Chi image for β = 45°. The illustration shows the structural color images under the corresponding observation direction. Figure S8: Quantitative randomness evaluation of PUF information, according to different structural orientations of Tai Chi image for β = 35° (a, b), β = 25° (c, d) and β = 15° (e, f). Figure S9: Structural color images of samples treated with toluene and xylene solutions. Figure S10: Multi-channel information(a), three-dimensional color space distribution (b) and the corresponding distributions of brightness and saturation (c), PMF—Correlation Coefficient(d), PMF—Hamming Distance(e) and Entropy—Sample Number(f) of the trademark image at time t0. Figure S11: Multi-channel information(a), three-dimensional color space distribution (b) and the corresponding distributions of brightness and saturation (c), PMF—Correlation Coefficient(d), PMF—Hamming Distance(e) and Entropy—Sample Number(f) of the trademark image at time t1. Figure S12: Multi-channel information(a), three-dimensional color space distribution (b) and the corresponding distributions of brightness and saturation (c), PMF—Correlation Coefficient(d), PMF—Hamming Distance(e) and Entropy—Sample Number(f) of the trademark image at time t2. Figure S13: Blue circular picture(a), three-dimensional color space distribution (b) and the corresponding distributions of brightness and saturation (c), PMF—Correlation Coefficient(d), PMF—Hamming Distance(e) and Entropy—Sample Number(f) of the blank group.
