# Supplementary material for: Flexible and Physically Unclonable Function Anti-Counterfeiting Labels via Multi-Level Dynamic Structural Color Encryption
Source: Materials (Basel). 2026 Apr 2;19(7):1428. doi: 10.3390/ma19071428 (PMC13075175; doi:10.3390/ma19071428)
Supplement: Supplementary file 1 [file materials-19-01428-s001.zip › materials-4212637-supplementary.pdf]

Supporting information for

Flexible and physically unclonable function anti-counterfeiting labels via multi-level dynamic structural color encryption

Junzhe Lin [1], Min Zhao [1], Xueqing Zhu [1], Ruohan Guo [1], Dan Guo [1], Tianrui Zhai [1,*]

[1]Department of Physics and Optoelectronic Engineering, Beijing University of Technology, Beijing 100124, China

*Correspondence: trzhai@bjut.edu.cn

Corresponding Authors

*E-mail: trzhai@bjut.edu.cn

Supplementary Figures

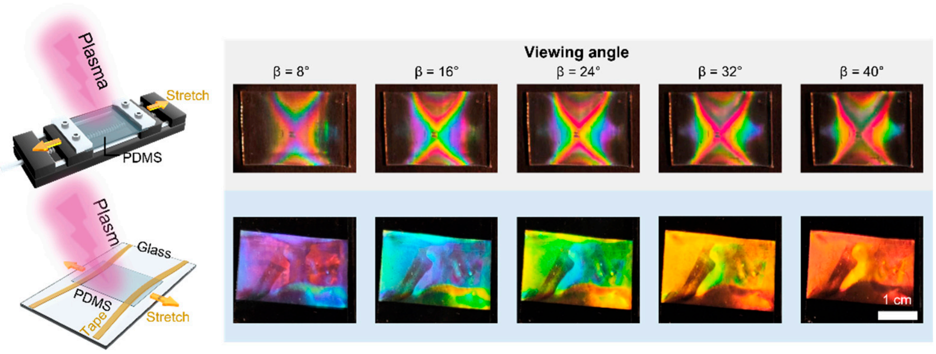

Figure S1. Schematic diagrams and structural color images of the two fabrication processes.

As illustrated in Figure S1, when the flexible film is uniformly stretched from both sides, the resulting micro-nano structures exhibit a well-defined, patterned arrangement due to the Poisson effect. While this ordered morphology can be reproduced consistently, it does not inherently introduce physically unclonable function (PUF) characteristics and therefore offers limited uniqueness for anti-counterfeiting applications. In contrast, the tape-assisted random stress-release process not only preserves the formation of ordered nanostructures but also introduces a high degree of randomness. This randomness, stemming from stochastic stress-release points, effectively embeds a PUF into the structural color label, significantly enhancing its anti-counterfeiting and encryption performance.

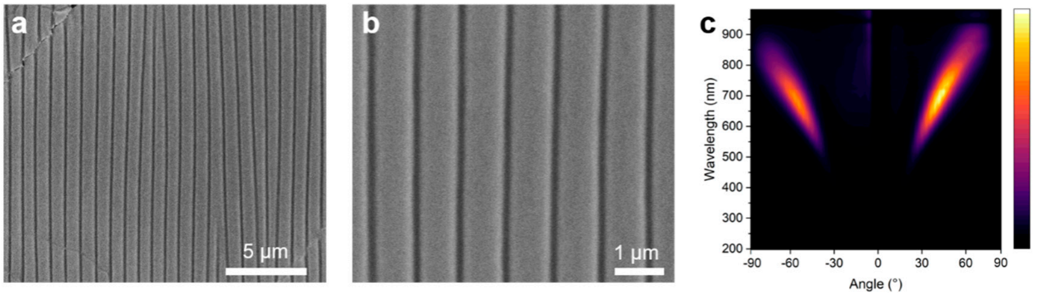

Figure S2. (a-b) SEM images of PCPUL. (c) Angle-resolved spectroscopy.

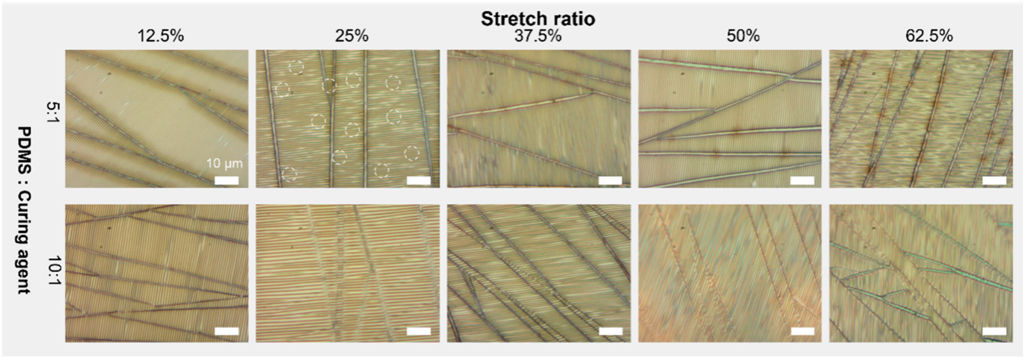

Figure S3. Microscope images of samples prepared with different stretching ratios and polymer compositions. Scale bar is 10 μm.

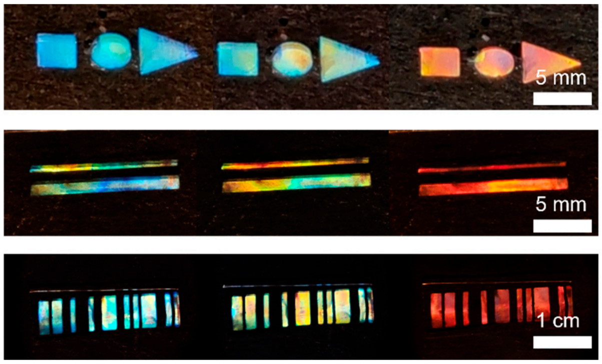

Figure S4. Structural color images of different graphic elements.

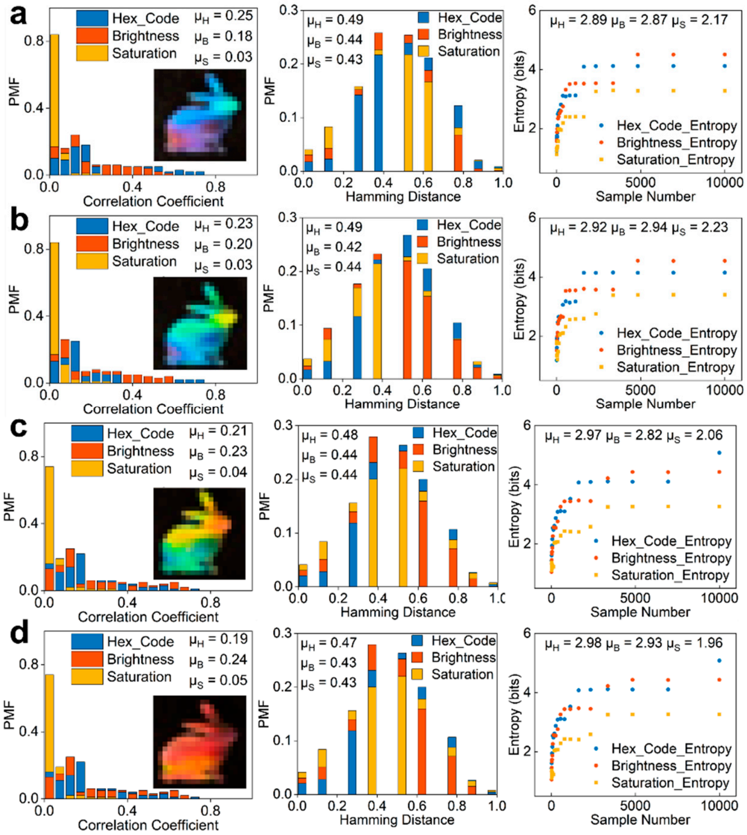

Figure S5. Randomness assessment of color information in the rabbit image for β = 15°(a), β = 25°(b), β = 35°(c) and β = 45°(d).

As shown in Figure S5, in the randomness assessment, the μ value of ideal random data has specific expectations. The Hamming distance μ = 0.5, the correlation coefficient μ = 0, and the entropy value μ = 8. Based on the correlation coefficient histogram (PMF - Correlation Coefficient), we can observe that the data distribution is concentrated around 0. The corresponding μ value range is $0.19 < \mu_H < 0.25$, $0.18 < \mu_B < 0.24$ and $0.03 < \mu_S < 0.05$. The closer μ is to 0, the better the randomness. Similarly, based on the PMF - Hamming Distance, the distribution should be symmetrical around 0.5, with the μ value close to 0.5. If the distribution leans towards 0 or 1, it indicates that the data are either very similar or have excessive differences, and the randomness is

poor. We can observe that the Hamming distance demonstrates that the sample data possess excellent randomness. However, according to Entropy - Sample Number, μ_H, μ_B and μ_S can only prove that the sample has a relatively high entropy value. Based on the analysis of these three parameters, the sample demonstrates good randomness.

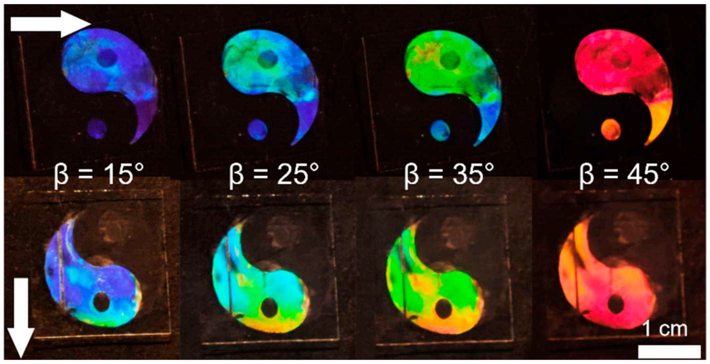

Figure S6. Structural color images from different observation directions.

Figure S6 demonstrates the angle-dependent retrieval of distinct structural color information along two perpendicular directions. Furthermore, to accommodate varying user needs, our fabrication process enables the extraction of patterned information from multiple observation angles.

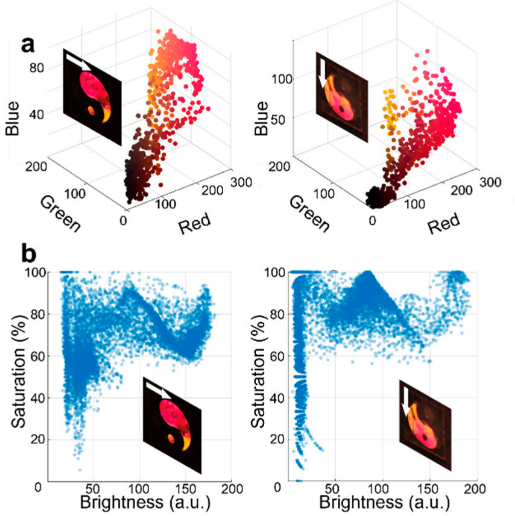

Figure S7. Three-dimensional color space distribution (a) and the corresponding distributions of brightness and saturation (b) of Tai Chi image for β = 45°. The illustration shows the structural color images under the corresponding observation direction.

As shown in Figure S7, although the two images appear similar color, there are still significant differences in their color three-dimensional distribution and brightness - saturation distribution at the details. This determines that they can both be used as encryption matrices in the encrypted database.

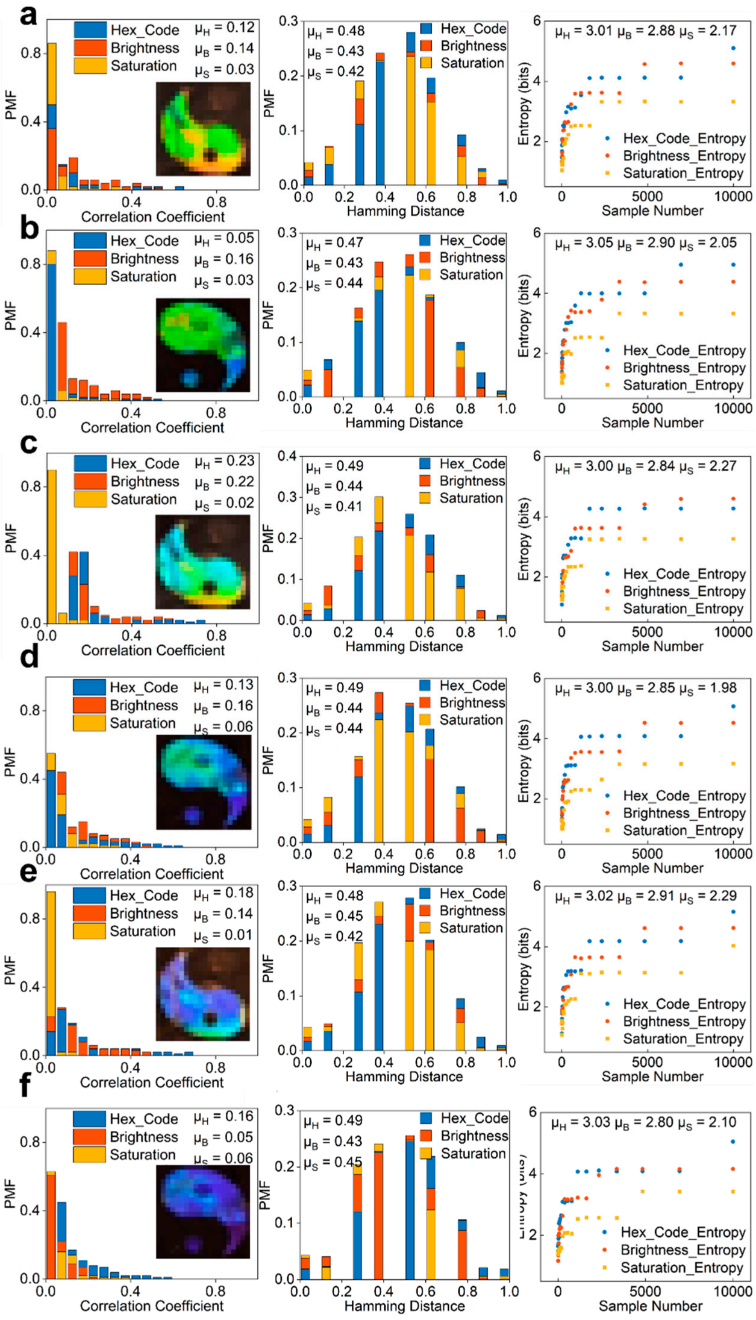

Figure S8. Quantitative randomness evaluation of PUF information, according to different structural orientations of Tai Chi image for β = 35° (a, b), β = 25° (c, d) and β = 15° (e, f).

As shown in Figure S8, the randomness of the Tai Chi image is better than that of the rabbit image, which means that the pattern process of the Tai Chi provides PCPUL with stronger non-clonability.

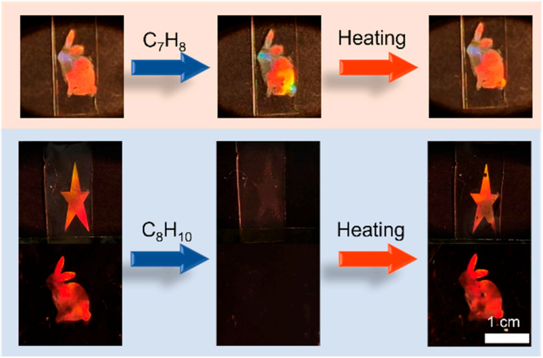

Figure S9. Structural color images of samples treated with toluene and xylene solutions.

As shown in Figure S9, exposure of the patterned flexible polymer film to C_7H_8 (toluene, boiling point 111 °C) induces swelling and deformation of the micro-nano structures. By controlling the solvent dosage, localized color changes or even complete disappearance of the structural color can be achieved. We also examined xylene (boiling point 138 °C), which exhibits a stronger swelling effect, though it may lead to irreversible structural damage. These results demonstrate that the dynamic encryption sequence can be tuned by selecting the solvent type and regulating its dosage. Furthermore, the recovery kinetics of the structural color can be modulated by applying mild heating, offering a versatile approach for enhancing the security of anti-counterfeiting codes.

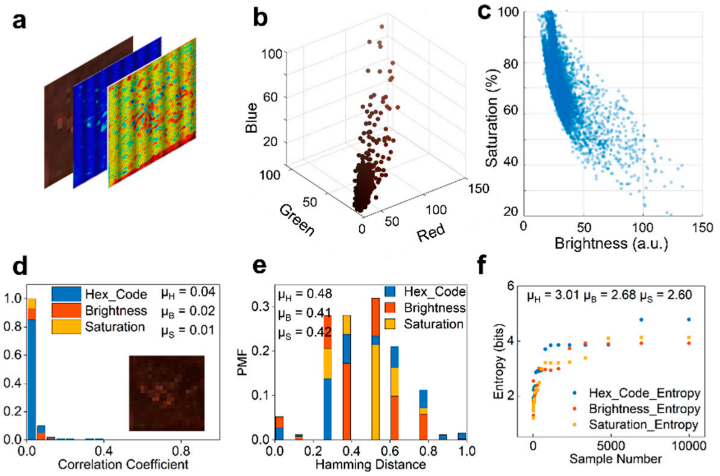

Figure S10. Multi-channel information(a), three-dimensional color space distribution (b) and the corresponding distributions of brightness and saturation (c), PMF - Correlation Coefficient(d), PMF - Hamming Distance(e) and Entropy - Sample Number(f) of the trademark image at time t_0.

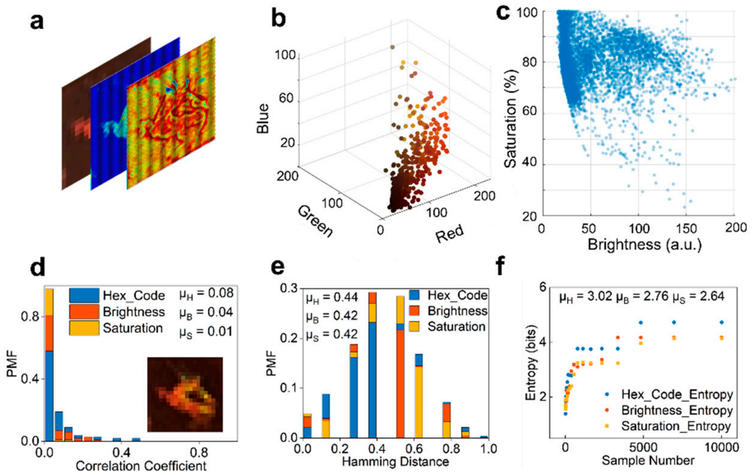

Figure S11. Multi-channel information(a), three-dimensional color space distribution (b) and the corresponding distributions of brightness and saturation (c), PMF - Correlation Coefficient(d), PMF - Hamming Distance(e) and Entropy - Sample Number(f) of the trademark image at time t_1.

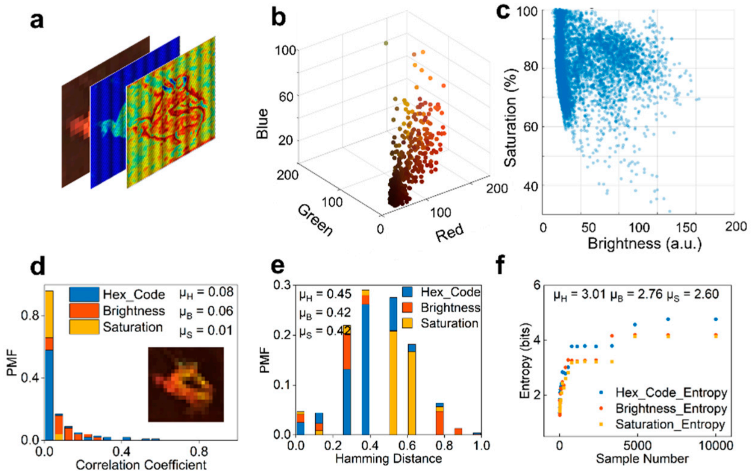

Figure S12. Multi-channel information(a), three-dimensional color space distribution (b) and the corresponding distributions of brightness and saturation (c), PMF - Correlation Coefficient(d), PMF - Hamming Distance(e) and Entropy - Sample Number(f) of the trademark image at time t_2.

As shown in Figures S10 - S12, the trademark patterns with complex contours perform the best in terms of randomness assessment, and this conclusion is applicable to every frame image in the time series.

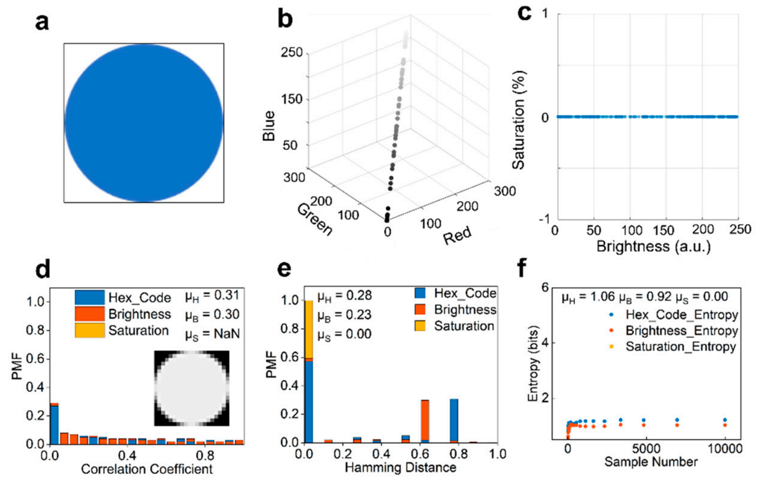

Figure S13. Blue circular picture(a), three-dimensional color space distribution (b) and the corresponding distributions of brightness and saturation (c), PMF - Correlation Coefficient(d), PMF - Hamming Distance(e) and Entropy - Sample Number(f) of the blank group.

As shown in the Figure S13, we selected a pure blue circular picture to calculate the blank group data. Considering the boundary effects caused by the limited sequence length and the noise and imperfection of the real data. Therefore, under the conditions of this study, we found that the threshold for strong numerical correlation: for PMF - Correlation Coefficient, $\mu > 0.3$; for PMF - Hamming Distance, $\mu < 0.35$; for Entropy - Sample Number, $\mu < 1$. And this threshold still has great potential for optimization, which requires a larger amount of data and more powerful computing capabilities.
